# Reduced knee joint loading with lateral and medial wedge insoles for management of knee osteoarthritis: a protocol for a randomized controlled trial

**DOI:** 10.1186/1471-2474-15-405

**Published:** 2014-12-03

**Authors:** Ryan T Lewinson, Kelsey H Collins, Isabelle A Vallerand, J Preston Wiley, Linda J Woodhouse, Raylene A Reimer, Jay T Worobets, Walter Herzog, Darren J Stefanyshyn

**Affiliations:** Schulich School of Engineering, University of Calgary, Calgary, Alberta Canada; Faculty of Kinesiology, University of Calgary, Calgary, Alberta Canada; Faculty of Medicine, University of Calgary, Calgary, Alberta Canada; Faculty of Rehabilitation Medicine, University of Alberta, Edmonton, Alberta Canada; Human Performance Laboratory, University of Calgary, 2500 University Drive NW, Calgary, Alberta T2N 1 N4 Canada

**Keywords:** Footwear, Insoles, Orthotics, Knee adduction moment, Pain

## Abstract

**Background:**

Knee osteoarthritis (OA) progression has been linked to increased peak external knee adduction moments (KAMs). Although some trials have attempted to reduce pain and improve function in OA by reducing KAMs with a wedged footwear insole intervention, KAM reduction has not been specifically controlled for in trial designs, potentially explaining the mixed results seen in the literature. Therefore, the primary purpose of this trial is to identify the effects of reduced KAMs on knee OA pain and function.

**Methods/design:**

Forty-six patients with radiographically confirmed diagnosis medial knee OA will be recruited for this 3 month randomized controlled trial. Recruitment will be from Alberta and surrounding areas. Eligibility criteria include being between the ages of 40 and 85 years, have knee OA primarily localized to the medial tibiofemoral compartment, based on the American College of Rheumatology diagnostic criteria and be classified as having a Kellgren-Lawrence grade of 1 to 3. Patients will visit the laboratory at baseline for testing that includes dual x-ray absorptiometry, biomechanical testing, and surveys (KOOS, PASE activity scale, UCLA activity scale, comfort visual analog scale). At baseline, patients will be randomized to either a wedged insole group to reduce KAMs, or a waitlist control group where no intervention is provided. The survey tests will be repeated at 3 months, and response to wedged insoles over 3 months will be evaluated.

**Discussion:**

This study represents the first step in systematically evaluating the effects of reduced KAMs on knee OA management by using a patient-specific wedged insole prescription procedure rather than providing the same insole to all patients. The results of this trial will provide indications as to whether reduced KAMs are an effective strategy for knee OA management, and whether a personalized approach to footwear insole prescription is warranted.

**Trial registration:**

NCT02067208.

**Electronic supplementary material:**

The online version of this article (doi:10.1186/1471-2474-15-405) contains supplementary material, which is available to authorized users.

## Background

Arthritis is one of the most prevalent musculoskeletal disorders, currently affecting 15% of people aged 15 years and older (~4.6 million Canadians), with an economic cost of $33 billion annually in Canada [1, 2]. The most common form is knee osteoarthritis (OA), affecting 16% of adults over the age of 45 [3]. It is characterized by degeneration of the knee joint cartilage, leading to severe pain, stiffness and swelling [4]. Commonly, symptoms will be severe enough to cause long-term disability [5, 6]. Currently, the gold standard treatment for severe OA is total joint arthroplasty (TJA) [7]. However, the majority of OA patients may be more suited for conservative management of OA, to either minimize symptoms in the short term, or ultimately prevent progression of OA to avoid the need for TJA.

Attention has been directed toward biomechanical interventions as they are generally low cost and relatively easy to implement. Biomechanically, cross-sectional and prospective studies have suggested that knee OA development and progression are related to increased peak external knee adduction moments (KAMs) during the single-leg support phase of walking [4–6]. This increased KAM is believed to induce large stress in the medial compartment of the knee, which, over time, contributes to cartilage degeneration, pain and ultimately OA [8]. Supporting this concept, increased KAMs have been positively correlated with higher Kellgren Lawrence grades, and cartilage loss over 12 months (KAM impulses), and negatively correlated with tibial cartilage thickness [5, 6, 9]. Thus, a focus for knee OA prevention and management has been to reduce these KAMs [4].

Across 13 international clinical guidelines for management of knee OA, wedged insoles (i.e. insoles that elevate either the lateral or medial aspect of the foot) have been consistently recommended for disease management since they can influence KAMs [10]. On average across patients, laterally wedged insoles have been shown to reduce KAMs and reduce pain, while medially wedged insoles tend to increase KAMs; [4, 11] however, when considering subject specific reactions, 15-43% of patients, depending on the study, receiving either a medial or lateral wedge will experience KAM changes that are completely opposite to the group average result [12, 13]. This means that every year, thousands of North Americans with knee OA are being prescribed an incorrect wedge resulting in increased KAMs, and possibly accelerating and worsening their OA.

Interestingly, when reviewing past literature of major randomized trials for knee OA, no study has actually ensured that all patients experienced a reduction in KAMs in the experimental insole group, and this has likely had a large bias on study results [14, 15]. Thus, it is no surprise that a recent review has highlighted that large variability exists across studies in terms of the effectiveness of lateral wedge insoles [16]. Essentially, we still do not truly understand how or if wedged insoles and reduced KAMs can benefit patients with knee OA. Therefore a randomized trial is urgently needed that assigns wedged insoles on a personalized basis to ensure that KAMs are being reduced. Thus, the primary purpose of this study is to evaluate the effectiveness of a personalized wedged insole intervention on pain in patients with medial knee OA. Specifically, KAMs must be measured for each participant to ensure KAM reduction occurs with the intervention insole, rather than providing all patients with the exact same insole and assume a constant KAM effect as previous trials have done. This approach of ensuring KAM reduction has never been attempted before and so the results from this study will help evaluate the true clinical benefit of KAM reduction, and also the true effectiveness of wedged insoles. It is hypothesized that patients in the experimental group, where KAMs have been reduced using an individually prescribed wedged insole, will overall have greater improvements in pain and physical function over 3 months compared to the control group.

## Methods

### Participant consent & ethical approval

Ethics approval for this study has been obtained from the University of Calgary Conjoint Health Research and Ethics Board (CHREB). In accordance with CHREB guidelines, written consent will be obtained from each individual prior to being enrolled in the study formally as a participant, and prior to any testing.

### Eligibility, recruitment and evaluation for inclusion

**Figure 1:**
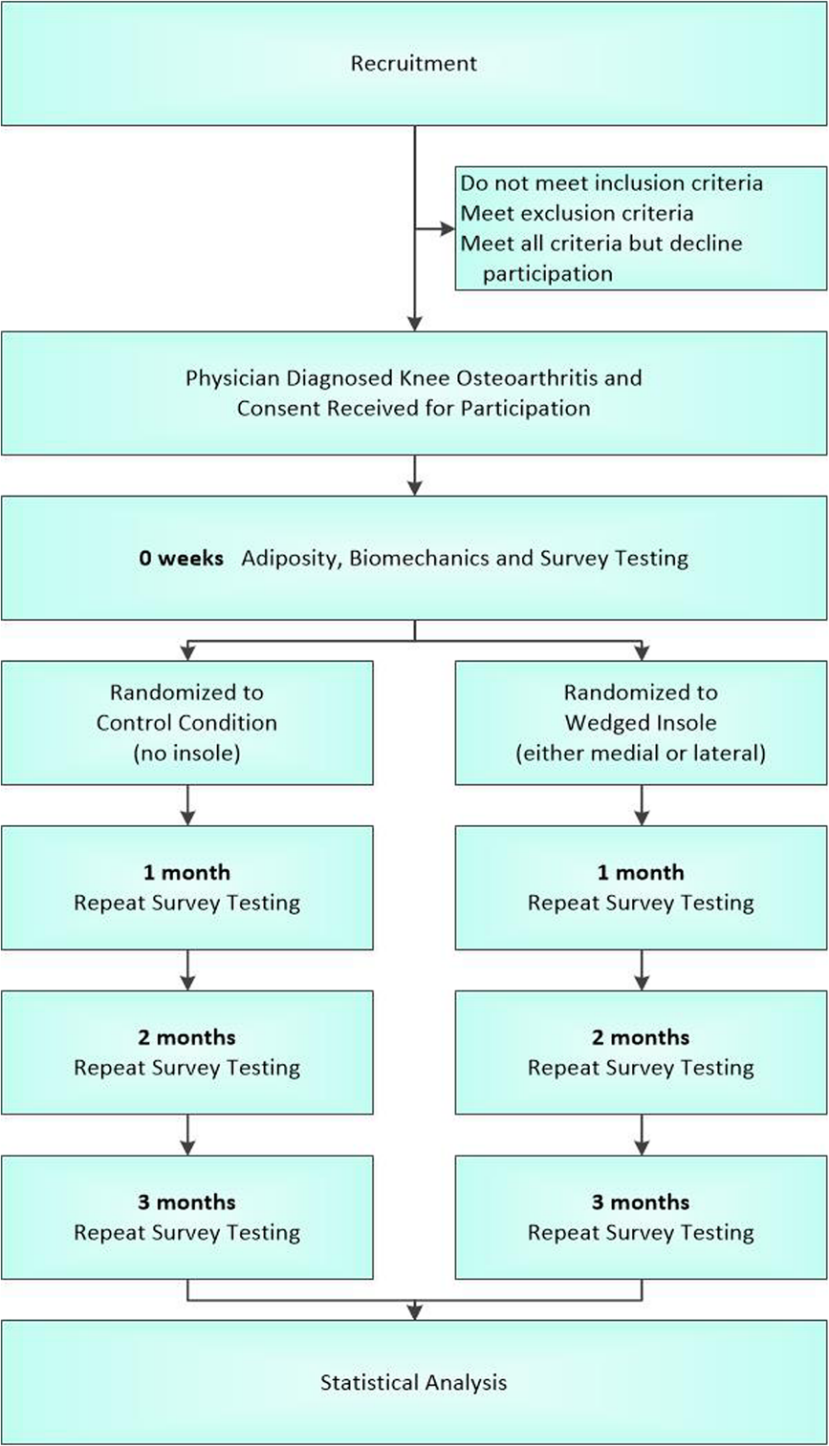
**Flow of participants through the study.**

The trial will have two parallel groups, where participants are randomly assigned to a waitlist control or experimental arm for a study duration of 3 months (Figure 1). Patients with painful radiographic medial knee osteoarthritis will be recruited from either the University of Calgary Sport Medicine Centre patient population (a Knee Osteoarthritis clinic is part of the services provided by this centre) or the Alberta Hip and Knee Arthroplasty clinics. Identified patients will be approached for inclusion into the study based on the inclusion/exclusion criteria described below. If recruitment is too slow to meet study timelines, advertisements of the project will be put into local newspapers and to other media. In these cases, a phone assessment of the volunteer will be undertaken for preliminary screening, and the individual will be invited for a detailed assessment by the study physician to determine their eligibility.

All patients considered eligible for the study must have physician-diagnosed knee OA primarily localized to the medial tibiofemoral compartment, based on the American College of Rheumatology clinical and radiographic diagnostic criteria [17]. Patients showing lateral compartment or patellofemoral OA in addition to medial knee OA will still be considered eligible as long as the medial compartment is the primary disease location and symptoms are attributed to the medial compartment. In addition, patients must be between the ages of 40–85 years of age, have no history of viscosupplement injections within the past 6 months, cortisone injection within the past 3 months, narcotic pain medication within the past 3 months, or use of other footwear insoles or unloader knee braces within the past 2 months, and must have a Kellgren-Lawrence grade (a measure of OA severity) of 1 to 3, as determined by a clinician, to avoid inclusion of end-stage OA patients that may be more appropriate for surgery [18]. Patients may continue to use anti-inflammatory medication or acetaminophen for mild pain management if needed. Those who meet the intrarticular injection criteria must also be willing to not undergo further treatment during the 3-month study. Patients must also present with a KOOS pain subscale score of 75 points or lower. This will help ensure that all participants have at least mildly painful knee OA. Any participant with recent (within 2 months) musculoskeletal or neuromuscular injury to the lower extremity that could bias pain assessments or gait analysis will not be considered eligible. For this reason, patients who typically use a cane will also be excluded.

### Footwear insole interventions

Two footwear insole interventions will be utilized within the experimental arm of this study: (1) the first is a medial wedge insole placed within the participants own shoe. The insole will be developed using a 3D printer, where the wedge thickness is 0.5 mm on the lateral edge, and 6.5 mm on the medial edge. The insole will taper at the metatarsal heads to a thickness of 4.5 mm to ensure a comfortable fit within the participant’s shoe. A wedge thickness of about 6 mm has been used previously and has been shown to be large enough to influence knee mechanics, yet small enough to maintain comfort [14, 19–21]. During testing and long term use, this insole will be placed under the existing sock liner of the participant’s shoe. (2) The second condition is a lateral wedge insole placed within the participants own shoe. The insole will be developed using a 3D printer, where the thickness of the medial edge is 0.5 mm and the thickness of the lateral edge is 6.5 mm, tapering to 4.5 mm beyond the metatarsal heads. During testing and long term use, this insole will be placed under the existing sock liner of the participant’s shoe. For construction of the medial and lateral wedge, the wedge will extend along the full length of the foot, creating approximately 5–6 degree medial and lateral wedges [22]. All insoles will be produced by New Balance Athletic Shoe Inc. (Boston, MA). The material utilized for the insoles is stiff in compression, such that the wedge shape will be maintained throughout the 3 month study; however, the material is also flexible to ensure comfort within the shoe during flexion of the metatarsal joints.

The authors must emphasize the relationship between the intervention we intend to utilize (wedged insoles), and the mechanical effect we aim to achieve (reduced KAMs). It is this mechanical result (reduced KAMs) that is suggested in the literature to affect knee OA – not wedged insoles on their own. Thus it is critical to bear in mind that the wedged insoles are simply a means to achieve the desired mechanical outcome.

Recently, it has been reported by a meta-analysis that future knee OA trials should utilize a flat insole as a control condition [16]. The motivation for this claim was based on the notion that this would control for placebo effects. However, recent evidence has suggested that even flat insoles can alter pressure distributions under the foot during gait [23], and thus it remains a possibility that KAMs could be influenced as well. Therefore, while flat insoles may control for placebo effects, they may not control for gait biomechanics, which is the primary concern of our trial. Consequently, the control condition for our study will be the participant’s own footwear, as this will control for gait biomechanics changes. Therefore, for biomechanical reasons, the trial will not be placebo controlled. In effort to ensure recruitment, compliance and participant retention in our control condition, we will utilize a waitlist control strategy, where we will indicate that a 3 month wait is required prior to providing an insole. After 3 months, we will provide control participants with access to a wedged insole, and continue to monitor them for safety.

### Laboratory testing protocol

All eligible participants will be invited to the laboratory for baseline testing. At baseline, body metrics such as height (m), mass (kg) and age (years) will be recorded, and also basic clinical diagnostic data (eg. Kellgren-Lawrence grade).

#### Body composition

Lean and fat mass will be evaluated at baseline using Dual Energy X-Ray Absorptiometry (DXA, Hologic QDR 4500; Hologic, Bedford, MA), the clinical gold standard for body composition.

#### Biomechanics

Eight Motion Analysis cameras (Motion Analysis Corp., Santa Rosa, CA) sampling at 240 Hz will record the 3D trajectories of retroreflective markers placed over the participants most symptomatic limb as they walk along a 20 m runway. A force platform (Kistler AG, Winterthur, Switzerland) will collect ground reaction force data at a frequency of 2400 Hz as the participant walks along the runway. This will be done for 5 successful trials in each of 3 footwear conditions for each participant: (1) participant’s own shoe (baseline condition), (2) participant’s own shoe with the lateral wedge insole, and (3) participant’s own shoe with the medial wedge insole. A successful trial is defined as one where the participant lands with their most symptomatic limb near the center of the force platform, does not touch the force platform with their other limb, and maintains a gait speed of 1.3 ± 0.07 m/s as measured by two photocells.

Kinetic and kinematic data will be filtered using 4^th^ order Butterworth low-pass filters with cut-off frequencies of 50 Hz and 12 Hz, respectively. KAMs during stance will be calculated using an inverse dynamics approach, and the peak KAM (first peak) will be identified for each trial [4, 19]. The average KAM across trials for each footwear condition will then be calculated and represented as a percent change from the baseline condition value. The KAM impulse (integral of KAMs with respect to time) and Varus thrust (maximum mediolateral displacement of knee joint center from touchdown) [24] and the KAM impulse (integral of KAMs with respect to time) [25] will also be calculated as these variables have also been shown to be related to knee OA [6, 24]. At the baseline visit, biomechanical testing and DXA scans will be performed and survey information will be collected. After determining the peak KAMs for all three conditions, the percent change in KAM will be determined for the medial and lateral wedge conditions.

### Survey protocol

#### Patient history

Detailed musculoskeletal injury histories will be taken for each participant. This will be done on a recall basis, and will specifically request that patients outline any previous traumatic, acute knee injuries (e.g. meniscal injury, patellofemoral injury, proximal tibial or distal femoral fractures, plica, knee ligament damage including avulsion tears, or any large impacts to the knee causing excessive bruising or swelling), surgical procedures on the knee, or any history of heavy loading activities (e.g. American football, running, ski jumping etc.).

#### KOOS

The Knee Injury and Osteoarthritis Outcomes Score (KOOS) is based on the Western Ontario and McMaster Universities (WOMAC) Arthritis Index– a survey that is both valid and reliable for measurement of pain, function and stiffness in OA populations [26–32]. Additionally, the KOOS includes subscales that can evaluate function in activities of daily living and during more rigorous activity, allowing it to be sensitive to differences in activity between individuals. The five KOOS components include: pain, symptoms, function in daily living, function in sport and recreation, and knee related quality of life. The KOOS has been extensively validated and can be used over long periods of time, allowing for possibility of long-term follow-up of the recruited patient cohort [33–37]. The KOOS will be administered at baseline, and at 1 month, 2 months and 3 months follow-up.

#### Physical activity

Two physical activity scales will be used in this study. The first is the UCLA physical activity scale. This scale provides a global measure of activity, and is commonly used in osteoarthritis populations undergoing arthroplasty [38]. The second is the Physical Activity Scale for the Elderly (PASE). PASE will be used to evaluate the type and duration of recreational and occupational physical activity of the previous week [39]. This scale was originally developed and validated for individuals over the age of 55 years, which is appropriate for the majority of the knee OA population. The purpose of the physical activity scales are to identify whether physical activity levels generally increased, decreased or remained constant during the study for experimental and control participants. The tests will be administered at baseline, and at 1 month, 2 months and 3 months follow-up.

#### Adherence

At baseline, participants will be asked to refrain from utilizing any alternate therapies during the study period (aside from anti-inflammatory or acetaminophen medication), and asked to utilize the prescribed footwear condition as frequently as possible during the day. While not an exclusion criteria of this study, participants will be asked to try and always use the insole in the same shoe tested at baseline. We will conduct a survey at 1 month, 2 months and 3 months follow-up to evaluate participant adherence to the intervention, which will include recall of the number of hours per day using the assigned insole over the past week, recall of the number of times in the past week a different shoe from baseline was used with the insole, and recall of the number of times alternate allowable therapies were used, such as targeted exercise for OA or anti-inflammatory and acetaminophen medications. Use of therapies that are considered as exclusion criteria (i.e. viscosupplementation, knee braces, narcotic pain medication), will also be recorded.

#### Comfort

Some studies have suggested that footwear comfort may be an important variable in determining the effectiveness of a footwear intervention on pain management [40]. Therefore, we will administer a paper-based 100 mm visual analog scale with the terms “extremely comfortable” and “extremely uncomfortable” as anchors at 100 mm and 0 mm, respectively. The tests will be administered on paper at baseline.

### Randomization

Following the baseline laboratory and survey testing, participants will be randomized into one of two study arms: (1) a KAM-reduction experimental group (KAM-R), or (2) a waitlist control group. In the KAM-R group, participants will be given either a medial or lateral wedge insole (whichever insole reduces KAMs more for each individual participant) to put inside their own shoe and use for 3 months. In the waitlist control group, participants will be asked to continue using their own shoes for a period of 3 months, and following this will be provided with access to a wedged insole (whichever reduces KAMs more based on baseline data collection).

Participants will be randomized using a multi-block-randomization procedure into either the KAM-R or waitlist control group. Specifically, a separate block sequence will be used for males and females to ensure an even sex distribution across groups. A block size of 6 will be used for each, and the sequence will be determined from a random number generator.

The one exception to this block randomization procedure is that if neither wedged insole produces a KAM reduction, the participant will be excluded from the main study altogether. This scenario will not be common, as most people do experience a large change in KAMs with one of the two wedged insole types, but this strategy will ensure that all participants in the KAM-R and waitlist control groups are all biomechanically suitable for a wedged insole intervention. Thus, neither group will contain biomechanical non-responders to wedged insoles.

### Blinding

Knowledge of the participant’s KAM change is required to ensure that the study sample consists entirely of those who experience a reduction in KAMs with at least one insole type. We will randomize patients to either the KAM-R or control group prior to knowing the KAM results and prior to any data collection. After collecting biomechanical data, KAM results will be assessed to verify that the participant experienced a KAM reduction in at least one wedged insole type. As described previously, in cases where the participant does not experience a KAM reduction with either insole, they will be excluded from the main study. Thus our knowledge of KAM changes will not affect our initial randomization, and only be used as a verification tool. Although researchers will be aware of each participant’s study group following randomization (in order to assign the participants in the KAM-R group an insole to take home), the researchers will be blinded during future survey data collection and data analysis as identifying information will be stripped by an independent researcher and replaced with unique patient ID numbers and numerical values for group allocation. Those involved in statistical analysis will be blinded to the group allocation and outcomes during data analysis. All KAM-R participants will be aware as to which condition they have been assigned, based on knowing that they have received an insole. All control condition participants will be aware that they have not received an insole at baseline, but will receive an insole at 3 months. Thus, the study will not be blinded at baseline data collection; however, follow-up data collection and data analysis will be blinded to the researchers.

### Primary outcome measures

The primary outcome measure in this study will be the KOOS pain score (0–100 points). This measure will allow us to identify the overall treatment effect of the wedged insole intervention over the 3-month follow-up period.

### Secondary outcome measures

Secondary outcome measures include the remaining four KOOS subsection scores (symptoms, function (daily living), function (sport & recreation activities), quality of life; 0–100 points each), PASE (0-400+ points) and UCLA (0–10 points) physical activity scores, peak KAM (Nm), KAM impulse (Nms), varus thrust (mm), adiposity (%), footwear comfort (0–100 mm) and the general intervention adherence survey. In addition, we will use Kellgren-Lawrence grade (grade 1–3) in secondary analyses.

### Sample size calculation

Sample size calculations were performed based on our primary outcome variable, the KOOS pain score, with a desired power of 80% and a significance level of 0.05 using the statistical software Stata version 11.2. Given the early stage of research concerning the effects and interactions of load reduction, and different OA subtypes on treatment outcome, we chose to only conduct sample size calculations on a repeated measures basis for within group comparisons. Based on the results of this trial, sample sizes may be expanded to increase statistical power in order to make comparisons between the control and KAM-R group.

The minimum perceptible clinical improvement and minimum detectible change in KOOS score has been reported to be about 13.4 points [33, 41]. Using a baseline KOOS pain score of 57.3 points [42] and a standard deviation of 15 points [41], to detect a minimal clinically important improvement of 13.4 points within each group, 20 participants are required in each group, or 40 total participants. To account for a potential dropout rate of 15%, we will recruit a minimum of 46 participants.

### Statistical analysis

Descriptive analyses will be performed on all variables. Outcome measures data will be plotted to assess trends of change over the 3 months.

#### Within group analyses

All data will be analyzed on an intention to treat basis. Changes from baseline to 3 months will be assessed within groups for ordinal data (UCLA score) using Wilcoxon Signed Rank test (α = 0.05). KOOS subsection scores and PASE scores will be compared between baseline and at 3 months using paired-samples t-tests (α = 0.05). KAMs, KAM impulse, varus thrust, and footwear comfort will be compared between the neutral shoe and assigned wedged insole condition at baseline for the KAM-R group using paired-samples t-tests (α = 0.05). All tests will be two-tailed, except in the case of KAMs and KAM impulse, where one-tailed tests will be used. The reason for this is that KAM reduction is strictly controlled in this study and so KAM and KAM impulse reductions are the only possible outcome.

A multivariable linear regression (α = 0.05) will also be conducted on the KAM-R group to evaluate whether KAM reduction, KAM impulse, varus thrust and footwear comfort can predict change in pain over 3 months. Covariates tested in these four models will include adiposity and Kellgren-Lawrence grade.

#### Between group analyses

Ordinal data from UCLA scores and Kellgren-Lawrence grades will be compared at baseline and at 3 months (UCLA score only) between the control and KAM-R group using Mann–Whitney U tests (α = 0.05). KOOS subsection scores, PASE scores, KAMs, KAM impulse, varus thrust, adiposity, footwear comfort, and adherence will be compared at baseline between the KAM-R and control group using a MANOVA test (α = 0.05) with independent-samples t-tests for post-hoc analysis. Changes over 3 months will also be assessed between groups using a MANOVA (α = 0.05) with independent-samples t-tests for post-hoc analysis. All tests will be two-tailed, except in the case of KAMs and KAM impulse, where one-tailed tests will be used. The reason for this is that KAM reduction is strictly controlled in this study and so a reduction in KAMs and KAM impulse is the only possible outcome. It should be noted that all between group comparisons are exploratory analyses, as we may not be powered to detect any significant differences between groups.

## Discussion

The primary goal of this randomized controlled trial is to identify the influence of reduced KAMs on management of knee OA symptoms. In this study, wedged footwear insoles will be used as the intervention to induce reduced KAMs. Wedged insoles are a relatively inexpensive and easy-to-use intervention, and have been a recommended management strategy in numerous international clinical guidelines for OA. However, some randomized trials conducted on wedged insoles have shown inconsistent results. The primary reason for this is that previous studies used exclusively a lateral wedge insole and did not test each participant’s biomechanics to ensure that the lateral wedge actually reduced KAMs. Indeed, it has been found in numerous studies that lateral wedges may in fact increase KAMs during gait for some participants, which could induce OA worsening, and wash out any actual treatment effects of reduced KAMs that may be observed in other participants [12, 13]. Thus, in our study, we are testing both medial and lateral wedge insoles, and prescribing the insole that reduces KAMs the most. This will ensure that all participants in our KAM-R group experience reduced KAMs.

Another reason why other wedged insole studies have shown mixed results on OA management is that these studies used inadequate control interventions. Specifically, some studies have given all control participants a flat neutral insole. Other studies have provided a new shoe to all participants as a control condition [40]. While in theory this is appropriate as an attempt to protect against placebo effects and provided a method for blinding, it does carry the possibility that some participants will experience altered KAMs during gait. For instance, McCormick et al. [23] have shown that control insoles alter pressure distributions relative to a no insole condition, and this pressure shift could affect KAM magnitude [4]. Therefore, in our study, we have chosen to utilize the participant’s own shoe as a control condition to ensure biomechanics remain unchanged. Our regression analyses will provide some insight into whether placebo effects do exist with wedged insoles. For instance, if the slope of the line of best fit is not near zero, it would suggest that change is pain is related to a reduction in KAM regardless of whether a placebo effect exists or not.

## Conclusions

In conclusion, this will be the first study to isolate the effects of reduced KAMs on symptom management for patients with mild to moderate medial knee OA over a follow-up period of 3 months. This study could therefore have strong clinical implications regarding personalized prescription of wedged insoles.

### Clinical relevance and importance

This randomized controlled trial will help provide insight to optimize clinical management strategies for patients with mild to moderate medial knee OA by evaluating the effects of a knee joint load-reducing intervention. These results could have immediate implications on clinical practice, specifically the efficacy of knee joint load-reducing insoles, and also help identify new areas of study for basic scientists.

## Authors’ information

RTL is a MD/PhD student in biomedical engineering. KHM is a PhD student in biomedical engineering. IAV is a PhD student in epidemiology. JPW is a practicing sport medicine physician and Associate Professor of Medicine and Kinesiology. LJW is an Associate Professor and David Magee Endowed Chair of Physical Therapy, Scientific Director of the Bone and Joint Health Strategic Clinical Network for Alberta Health Services, incoming President of the Canadian Physiotherapy Association, physical therapist and co-lead of the Alberta Team in Osteoarthritis. RAR is a Professor of Kinesiology, Biochemistry and Molecular Biology. JTW is an Adjunct Assistant Professor of Kinesiology. WH is a Professor of Kinesiology, Medicine and Engineering, Past President of the International Society of Biomechanics, and co-lead of the Alberta Team in Osteoarthritis. DJS is a Professional Engineer, Professor of Kinesiology and Engineering, and Associate Dean of Kinesiology.

## Authors’ original submitted files for images

Below are the links to the authors’ original submitted files for images. Authors’ original file for figure 1

## Abbreviations

DXA: Dual energy x-ray absorptiometry
KAM: Knee adduction moment
KAM-R: Experimental group of the study experiencing reduced KAMs
KOOS: Knee injury and osteoarthritis outcome score
OA: Osteoarthritis
PASE: Physical activity scale for the elderly
UCLA: University of California at Los Angeles physical activity scale.
