# Supplementary figures and images for: Reduced knee joint loading with lateral and medial wedge insoles for management of knee osteoarthritis: a protocol for a randomized controlled trial
Source: BMC Musculoskelet Disord. 2014 Dec 3;15:405. doi: 10.1186/1471-2474-15-405 (PMC4265477; doi:10.1186/1471-2474-15-405)

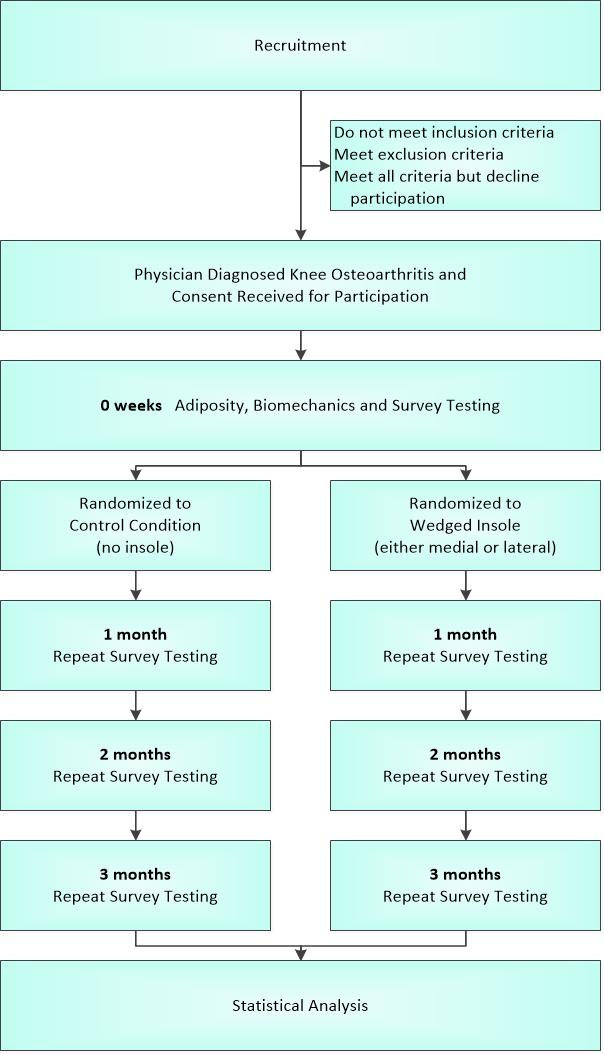

Supplement: Supplementary file 1 — Authors’ original file for figure 1 [file 12891_2014_2343_MOESM1_ESM.jpg]
